# Relationship Between Shift Work and Personality Traits of Nurses and Their Coping Strategies

**DOI:** 10.5539/gjhs.v8n5p166

**Published:** 2015-09-28

**Authors:** Fereshteh Farzianpour, Saeadeh Ansari Nosrati, Abbas Rahimi Foroushani, Fateme Hasanpour, Zahra Khakdel Jelodar, Meysam Safi Keykale, Mohammad Bakhtiari, Niusha Shahidi Sadeghi

**Affiliations:** 1 Department of Health Management and Economics, School of Public Health, Tehran University of Medical Sciences, Tehran, Iran; 2 Department of Epidemiology and Biostatistics, School of Public Health, Tehran University of Medical Sciences, Tehran, Iran; 3 Faculty of Health, Safety, and Environment, Shahid Beheshti University of Medical Sience, Tehran, Iran

**Keywords:** shift work, personality traits, nurse

## Abstract

**Background and Objective:**

Because of social progress, population growth, industrialization, and the requirements of some jobs, a significant percentage of employees are working in shifts. Shift work is considered a threat to health that could have unfavorable effects on various aspects of human life. This study investigated the relationship between shift work and the personality traits of nurses and their coping strategies in a selection of non-governmental hospitals in Tehran in 2014.

**Methods:**

This applied cross-sectional descriptive research employed the Standard Shift work Index and Eysenck Personality Questionnaire (EPQ) which, after confirmation of its validity and reliability (Cronbach’s alpha 0.73), were distributed among 305 nurses from 6 non-governmental hospitals in Tehran selected through cluster random sampling. Data was analyzed in two statistical levels: descriptive and inferential.

**Results:**

Results revealed that 43.6% of the nurses participating in the study were introverted and 56.4% were extroverted. There are significant relationships between age and physical health (P=0.008), sex and physical health (P=0.015), educational level and physical health (P=0.014), sex and cognitive, somatic anxiety (P=0.006), age and social-family status (P=0.001), marital status and social-family status (P=0.001), having a second job and social-family status (P=0.001), educational level and sleep and fatigue (P=0.002), work experience and coping strategies (P=0.044), and sleep and fatigue and personality traits (P=0.032).

**Conclusion:**

Complying with the standards of working hours for nurses and avoiding overtime when scheduling, especially for nurses with more work experience, can prevent the severe complications of shift work, enhance health, and ultimately enhance the quality of care. By improving the physical, psychological, and social health of nurses, the quality of patient care can be expected to improve, too.

## 1. Background

Because of social progress, population growth, industrialization, and the requirements of some jobs, a significant percentage of employees today are working in shifts (Mirmohammadi, 2010). Shift work is considered a threat to health that could have unfavorable effects on various aspects of human life (Positive Practice Environments, 2010).

Different types of shift work include fixed shifts, in which an individual typically works one shift (e.g., night shift), and unfixed shifts, in which an individual works different shifts and unscheduled working hours. On-call duty is a specific type of shift work in which a particular group of employees is called to work in emergency situations. The most widely-used shift system, however, is one in which the product or service is set in 8-hour shifts known as morning, evening, and night shifts (Mardi et al., 2014).

Several studies have investigated the negative effects of shift work on the performance, health, and quality of life of shift workers. Some of them have identified a relationship between shift work and the incidence of physical and psychological complications (Mahmodi et al., 2010). Shift work is more prevalent in large and complex workplaces and has significantly more adverse effects in such environments (Positive Practice Environments, 2010). The combination of shift work, multiple work environment risks, high physical and cognitive needs, reduced control of an individual over his own work, and other psycho-social stressors impose additional negative effects on the health of employees (Mehrabi et al., 2009).

One working group whose employees are shift workers because of the requirements of their jobs is health care workers, especially nurses (Mirmohammadi, 2010). In Iran, 80% of employees of the healthcare system are nurses (Suzuki et al., 2009). Nurses are at the forefront of providing health services, and they work all hours of the day and night to meet the needs of patients (Suzuki et al., 2009). Nurses work irregular morning, evening, and night shifts and are more exposed to psychological stresses than employees in other professions (Potter et al., 2010). Nurses are at risk for insomnia, and their irregular sleep-wake pattern results in a decrease in their sleep quality and duration as well as a decrease in their occupational performance (Samaha et al., 2007). Some other negative effects of shift work include a reduction in an individual’s tolerance for occupational and life stresses with a tendency towards an uncontrolled consumption of different drugs. Over 50% of night shift workers have sleep disorders (Salemy, 2010). There is a higher incidence of work accidents and errors and occupational burnout in night shift nurses than in nurses who work morning and evening shifts (Rajaratnam et al., 2011). For most nurses who work unfixed work shifts, working the night shift brings adverse complications and consequences to their normal life, many of which are uncontrollable. Shift work has negative physical, mental, and social effects on the personal lives of nurses, which can eventually impact their families as well. Moreover, long working hours endanger an individual’s health and safety (Machi et al., 2012). In addition to affecting the private life of every nurse, these negative complications cause a decrease in alertness and in occupational productivity, which can endanger the lives of patients under the service of this occupational group. Obviously, nurses who do not enjoy good general health cannot provide good care to their patients, such as physical and psychological support, and this increases the risk of occupational mistakes and accidents, the consequences of which ultimately impact the patient and the nurse (Anosheh et al., 2010).

Personality is an organized set of characteristics consisting of relatively stable and persistent traits which distinguish one individual from others (Shafie Abadi, 2010). Personality has different dimensions, and psychologists have presented different opinions about them. Among these opinions, Bernreuter’s theory states that personality has 6 aspects: neurotic tendencies, self-sufficiency, extraversion-introversion, dominance and submission, sociability, and confidence (Shafie Abadi, 2010).

Eysenck focused on the survey of samples, types of personalities, and their traits and pointed out a variety of psychological traits which, in psychology, are determined with titles such as traits, habits, and form. He believes that, based on the analysis of factors, 2 main dimensions of personality can be identified: Extraversion-Introversion and Neuroticism-Stability (Arruda et al., 2014).

The extraversion-introversion dimension of personality refers to traits representing a tendency to external or internal affairs. If an individual pays more attention to affairs outside themselves or their behavior is often caused by external factors, their personality is more inclined to extroversion, and if one’s behavior is often caused by internal factors and mental evaluation, his personality is inclined to introversion (Robbins Stephan, 2005). The owners of each personality aspect behave in specific manners, have specific expectations, have unique behavioral skills and abilities and different needs, and, based on their own personality patterns, have specific motivations, expectations, and goals. Moreover, for each type of personality, a different working environment is suitable (Narimani, 2010). Therefore, identifying an individual’s personality traits can make recruiting and maintaining them more desirable and improve their performance in an organization (Gari et al., 2009).

Another topic investigated in this research is coping strategies. In today’s world, stress is identified as one difficulty of human resources, and its damaging effects on the private and social lives of individuals are obvious. Employees of an organization use different coping methods in stressful situations (Haren et al., 2009).

To confront life events, having an awareness of personality factors is very important, because personality factors are closely related to how an individual sees the world and how one responds to stressful factors and situations (Grise, 2009).

By looking at the how some personality factors influence the use of coping strategies, and by understanding personality factors better, individuals’ coping strategies can be changed from maladaptive to adaptive mode, which will lead to their growth and development (Moonk et al., 2010).

Because of the importance of nurses’ health and the impact of shift work on shift workers’ physical and mental health, occupational performance, and safety on the one hand and the role of personality traits and coping styles in how nurses confront shift work on the other, this research investigated the relationship between the shift work of nurses and their personality traits and their coping strategies in a selection of non-governmental hospitals in Tehran.

## 2. Methods

The current descriptive-analytic study, performed in the non-governmental hospitals in Tehran city of Atiyeh, Mada’en, Pasteur, Mehrdad, Parsian, and Apadana, employed the two-stage cluster sampling method. First a list of all non-governmental hospitals in Tehran city was prepared. From that list, 6 hospitals were randomly selected, and then an equal number of nurses from each hospital were randomly selected. In non-governmental hospitals, work shifts are offered nurses on a fixed basis, i.e. morning, evening, and night shifts. In teaching and university hospitals, work shifts are offered on an unfixed basis; in one week, a nurse may work all three shifts. This change in the work system may disrupt nurses’ biological rhythms and their personal life schedules, and it could affect their responses to the study questions. Therefore, this type of shift work was excluded from the study, and sampling was carried out in nongovernmental hospitals only. The inclusion criterion was having worked in each fixed group for at least one year, so that the effect of work experience on the above-stated index could be measured. Because certain conditions inhibit an individual’s conformity to shift work and are considered to be confounding variables, nurses with a history of sleep disorders, asthma, diabetes, coronary artery disease, psychiatric disorders, epilepsy, gastrointestinal disorders, alcohol use, or drug abuse were excluded from the study.

Information on 30 clinical departments and the total number of the nurses in each of the afore-named hospitals was obtained through the hospital websites. The cluster random sampling method was chosen, and sample size was calculated as 305 subjects based on the correlation coefficient formula with a confidence level of 0.95 and a power of the test of 0.90.

The main tool for data collection in this research was a questionnaire comprised of three parts asking about demographic characteristics (age, sex, marital status, place of birth, educational level, work experience, work shift, type of overtime, employment status and second job), effects of shift work (sleep and fatigue, 5 questions; physical health, 20 questions; cognitive-somatic anxiety, 14 questions; social-family status, 18 questions; coping strategies, 6 questions), and personality traits (56 multiple-choice questions which measured the two main dimensions of personality, extroversion and neuroticism).

Using the paired t-test, the reliability coefficient of the questionnaire (Cronbach’s alpha) was calculated as 0.73. A previous study by Baraheni et al. verified the validity and reliability of the Eysenck Personality Questionnaire (EPQ) (Cronbach’s alpha 0.78). Data collected in this research was analyzed using SPSS V.22 software. Pearson’s correlation coefficient, the t-test, analysis of variance (ANOVA), the Mann-Whitney test, and the Kruskal-Wallis test were used to measure relationships between variables.

## 3. Results

Men comprised 25.6% of subjects (78 persons), and 74.4% were women (227 persons). 43.6% of subjects were single (133 persons), and 56.4% were married (172 persons). 10.5% of subjects had a high school diploma or an associate’s degree (32 persons), 81% had a bachelor’s degree (247 persons), and 8.5% had a master’s degree (26 persons). 76.4% of subjects had worked mandatory overtime (72 persons), and 23.6% had worked optional overtime (233 persons) (Table 1). Mean values of sleep and fatigue and social–family activity among nurses participating in the study were 38.52% and 23.37%, respectively. The maximum mean was related to sleep and fatigue. Given that these questions were negative; this mean reflects the unfavorable situation in this dimension. Higher scores for these variables of Cognitive-Somatic Anxiety, Coping Strategies indicate worse physical health; nurses’ physical health will develop a more undesirable status. Nurses are at risk for insomnia, and their irregular sleep-wake pattern results in a decrease in their sleep quality and duration as well as a decrease in their occupational performance. Obviously, nurses who do not enjoy good general health cannot provide good care to their patients, such as physical and psychological support, and this increases the risk of occupational mistakes and accidents, the consequences of which ultimately impact the patient and the nurse (Table 2).

**Table 1:** Frequency distribution of individuals completing the questionnaire, according to demographic variables

Variable	N (%)	Percent
**Gender**		
Male	78	25.6
Female	227	74.4
**Age**		
21-29 years	81	26.6
30-39 years	154	50.5
Older than 40 years	70	23
**Level of Education**		
High School Diploma or Associate’s Degree	32	10.5
Bachelor’s Degree	247	81
Master’s Degree or Higher	26	8.5
**Work Experience**		
Less than 5 years	88	28.9
5-10 years	101	33.1
Greater than 10 years	116	38
**Employment Status**		
Program or Temporary Contract	204	66.9
Long-Term Contract	57	18.7
Permanent	44	14.4
**Marital Status**		
Single	133	43.6
Married	172	56.4
**Work Shift**		
Fixed morning	83	27.2
Fixed evening	87	28.5
Fixed night	135	44.3
**Working Overtime**		
Mandatory	72	23.6
Optional	233	76.4
**Having a Second Job**		
Yes	95	31.3
No	101	68.8

**Table 2:** Mean different dimensions of shift work, in the nurses under the study

Dimension	Min	Max	Total	Mean	SD
Health Status	16	51	9461	31.12	7.105
Sleep and Fatigue	27	51	11672	38.52	4.585
Cognitive-Somatic Anxiety	14	39	7077	23.28	5.522
Social-Family Activity	12	40	7084	23.37	5.153
Coping Strategies	41	159	30254	101.52	21.501

Table 3 showed results of statistical analysis related to the relationship between demographic variables of the participants in the study and different dimensions of shift work.

**Table 3:** Results of statistical analysis related to the relationship between demographic variables of the participants in the study and different dimensions of shift work

Shift Work Dimensions	Sex	Age	Level of Education	Work Experience	Employment Status	Marital Status	Working Overtime	Having a Second Job	Work Shift
Physical Health Status	0.015	0.008	0.014	0.101	0.414	0.144	0.192	0.773	0.193

Cognitive - Somatic Anxiety	0.006	0.253	0.386	0.819	0.145	0.088	0.253	0.412	0.234

Social - Family Status	0.121	0.001	0.532	0.076	0.176	0.001	0.979	0.001	0.191

Sleep and Fatigue	0.326	0.555	0.002	0.095	0.443	0.826	0.371	0.771	0.059

Coping Strategies	0.146	0.307	0.909	0.044	0.388	0.569	0.910	0.565	0.850

Kolmogorov-Smirnov test was used to test the normality of physical health, cognitive-somatic anxiety, and social family status scores. Test Statistic=0.088 and

P<0.001 were obtained, indicating that the distribution of these scores is not normal; thus, the Spearman, Mann-Whitney, or Kruskal-Wallis correlation test was

used to examine the effects of demographic factors on the median of these scores. The Kolmogorov-Smirnov test was also used to test the normality of sleep and fatigue and coping strategies scores. Test Statistic=0.044 and P<0.05 were obtained, indicating the distribution of these scores is normal. Thus, the Spearman, Mann-Whitney, or Kruskal-Wallis correlation test was used to examine the effects of demographic factors on the median of these scores.

Results of the Mann-Whitney test examining the relationship between sex and cognitive-somatic anxiety suggest that the median score of this factor is lower in men than in women. In other words, cognitive-somatic anxiety in women is worse than that in men. One-way-ANOVA test results suggest that the mean difference of coping strategies scores in different work experiences are statistically significant. Scheffé’s post hoc test results suggest a significant difference between the mean scores of coping strategies in the two groups of between 5 and 10 years of experience and higher than 10 years.

According to the results shown in Table 4, the mean scores of sleep and fatigue and shift work of the two groups, extroverted and introverted, are significantly different P=0.03 (P<0.05). Results of the current study suggest that the score of the impact of personality traits on sleep and chronic fatigue is better in extroverted people than in introverted people. Therefore, identifying an individual’s personality traits can make recruiting and maintaining them more desirable and improve their performance in an organization.

**Table 4:** Relationship between nurses’ personality traits and the different dimensions of their shift work

Shift Work Dimensions	Group	N	Mean rank	Test result	
Physical Health Status	Introverted	132	155.45	Z = -0.513	
	Extroverted	172	150.24	P = 0.608	
Cognitive - Somatic Anxiety	Introverted	132	158.05	Z = -0.972	
	Extroverted	172	148.19	P = 0.331	
Social - Family Status	Introverted	132	146.75	Z = -0.918	
	Extroverted	171	156.05	P = 0.359	
	Group	Number	Mean	Standard deviation	Test result
Sleep and Fatigue	Introverted	131	39.17	4.483	T = 2.155
	Extroverted	172	38.03	4.614	P = 0.032
Coping Strategies	Introverted	131	100.71	22.320	T= -0.576
	Extroverted	172	102.15	20.890	P = 0.565

## 4. Discussion

The current study investigated the relationship between the shift work and the personality traits and coping strategies of nurses in a selection of non-governmental hospitals in Tehran in 2014. The results showed a significant correlation between age and physical health. As age increases (given that age questions are negative, higher scores for this variable indicate worse physical health), nurses’ physical health will develop a more undesirable status. A study by Imani et al. (2011) showed that the scores of general health were higher among younger nursing students.

Anna Korompeil et al. were first to use the Greek version to examine work shift standards. Their results showed that as nurses’ age increases, their physical health decreases; management should pay attention to this point in occupational planning (Korompeli et al., 2014).

Other results of the current study suggest that the median physical health score in men is significantly different from that in women. In other words, women have a worse physical health status than men. The results of Bazaziyan’s study are consistent with the results of the current study. The results of this study also showed that general health scores are higher in men than in women. Working different shifts, especially at night, can endanger the health status of female nurses who also undertake family responsibilities such as pregnancy and the upbringing of children (Bazaziyan et al., 2010).

The mean differences of sleep and fatigue scores indicate that the mean score of sleep and fatigue in the high school diploma and associate’s degree group differs from that in the bachelor’s degree group, and that of the high school diploma and associate’s degree group differs from that of the master’s degree group as well. Chehri et al. concluded in their study that there was a significant relationship between the educational level of high school diploma and higher and lack of sufficient sleep.

Other results of the study suggest that cognitive-somatic anxiety has a more undesirable status in women than in men. Zandi et al. found in their study that anxiety was higher in female nurses, and Korompeil et al. also concluded from their study that somatic anxiety was higher in female nurses (Korompeli et al., 2014).

Results from the current study suggest a significant correlation between social-family status and age. Therefore, it can be concluded that as age increases, nurses will have a more favorable social-family status. The median scores of social-family status in single and married people are different, with social-family status having a greater score in married nurses than in single nurses. Sharifzadeh’s study results showed a significant relationship between age and gender on one hand and work-family conflict on the other (Sharifzadeh et al., 2014).

Barnes et al. conducted their study in the four countries of Australia, Brazil, Croatia, and the United States and concluded that nurses were conflicted about establishing a balance between their occupational tasks and household tasks, and thus their working and familial lives were affected. They suggested that the work-family conflict can be reduced by reviewing and improving some work features, such as the length of work shifts, their duration and holiday work shifts (Barnes et al., 2010). Yu TAI et al. conducted their study on nurses aged 20-45 years in Taiwan. Their results suggested that family and social position had more favorable statuses in married nurses (Yu et al., 2014).

Examining coping strategies scores in different work experiences suggested that the mean differences are statistically significant. Scheffé’s post hoc test results indicate a significant difference between the mean scores of coping strategies in the two groups of between 5 and 10 years of experience and higher than 10 years of experience. The results of the SUE study are consistent with those of the current study. Several other studies have also suggested that when people are under occupational pressures and stresses, they apply a variety of coping strategies to eliminate or reduce the unpleasant effects of such pressures (SUE, 2010).

Results of the current study suggest that the score of the impact of personality traits on sleep and chronic fatigue is better in extroverted people than in introverted people. Rahimiyan et al. acheived similar results in their study (Rahimiyan et al., 2013).

## 5. Conclusion

Due to the harmful physical, psychological, and economic effects of shift work disorders on individuals and their organizational work place, efforts to reduce work pressure, review nurses’ tasks, review treatment and care programs in medical centers, better schedule working hours, and increase the number of nurses working in medical centers as well as preventive measures such as periodic physical examinations and consulting programs will be beneficial for employees (Rathore et al., 2012).

Considering the principles of circadian rhythms and applying them in hospital shift scheduling may help improve the nurses’ and patients’ health and safety statuses. In fact, by being aware of the effects of shift work and by taking measures to protect the health of employees, nurses and nursing managers can prevent professional and non-professional mistakes in their work place, which occur due to insomnia, fatigue, difficulty in focusing attention, and memory (Admi et al., 2010). Considering the fact that shifts work is inevitable in nursing, the search for ways to reduce the effects of it is necessary. The following strategies can be indicated: not scheduling night work for some people, such as nurses with medical conditions like depression, diabetes, or heart disease; providing opportunities for napping before the start of the shift to compensate for the lack of sleep as soon as possible; following a low-fat and high-fiber diet; exercising regularly if possible; avoiding being on-call for more than two consecutive nights; using the model of morning, evening, and night shifts for scheduling; changing shifts each 2 weeks (Martino I et al., 2013.).

Reducing the working hours of nurses and avoiding overtime, especially for nurses who have more work experience, can prevent occupational burnout and enhance the level of health and ultimately the quality of care. It can be expected that, by improving the physical, psychological, and social health of nurses, the quality of care given patients is also improved (Janssen et al., 2013).

Career guidance professionals assume that any kind of job selection and professional guidance requires an understanding of job characteristics and of individuals’ personality traits, interests, and backgrounds both by selectors and applicants. Otherwise, job-personality conflict results in an employee’s poor performance which may endanger the safety and welfare of others (Andrew, 2008).
